# Critical Role for Transglutaminase 2 in Scleroderma Skin Fibrosis and in the Development of Dermal Sclerosis in a Mouse Model of Scleroderma

**DOI:** 10.1002/art.43104

**Published:** 2025-05-19

**Authors:** Angela Y. Y. Tam, Korsa Khan, Shiwen Xu, Marianne Bergin, Linghong Huang, Erik Arroyo Colon, Danyi Cheng, Elisabetta A. M. Verderio, Voon Ong, Christopher P. Denton, John Atkinson, Tim S. Johnson, David J. Abraham

**Affiliations:** ^1^ University College London London United Kingdom; ^2^ UCB Slough United Kingdom; ^3^ Nottingham Trent University, Nottingham, United Kingdom, and University of Bologna Bologna Italy; ^4^ Present address: Mestag Therapeutics Limited, Cambridge, and University of Sheffield Sheffield United Kingdom; ^5^ Present address: Gilead Sciences Oxford United Kingdom

## Abstract

**Objective:**

Scleroderma is a life‐threatening autoimmune disease characterized by inflammation, tissue remodeling, and fibrosis. This study aimed to investigate the expression and function of transglutaminase 2 (TGM2) in scleroderma skin and experimentally induced dermal fibrosis to determine its potential role and therapeutic implications.

**Methods:**

We performed immunohistochemistry on skin sections to assess TGM2 expression and localization, and protein biochemistry of both systemic sclerosis–derived and healthy control dermal fibroblasts to assess TGM2 expression, function, and extracellular matrix deposition in the presence of TGM2 inhibiting and transforming growth factor (TGF)‐β neutralizing antibodies and a small‐molecule inhibitor of the TGF‐βRI kinase. Mice with a complete deficiency of TGM2 (*Tgm2‐/‐)* were investigated in the bleomycin‐induced model of skin fibrosis.

**Results:**

TGM2 was found to be widely expressed in both control and scleroderma skin samples, as well as in cultured fibroblasts. Scleroderma fibroblasts exhibited elevated TGM2 expression, which correlated with increased expression of fibrosis markers (Col‐1, αSMA, and CCN2). Inhibition of TGM2 using an inhibiting antibody reduced the expression of key markers of fibrosis. The effects of TGM2 inhibition were similar to those observed with TGF‐β neutralization, suggesting a potential crosstalk between TGM2 and TGF‐β signaling. Moreover, *TGM2* knockout mice showed significantly reduced dermal fibrosis compared with wild type mice. In vitro experiments with *TGM2*‐deleted fibroblasts demonstrated impaired cell migration and collagen matrix contraction, which could be partially restored by exogenous TGF‐β.

**Conclusion:**

TGM2 can regulate several key profibrotic activities of TGF‐β suggesting that attenuating TGM2 function may be of benefit in severe forms of connective tissue disease with skin fibrosis.

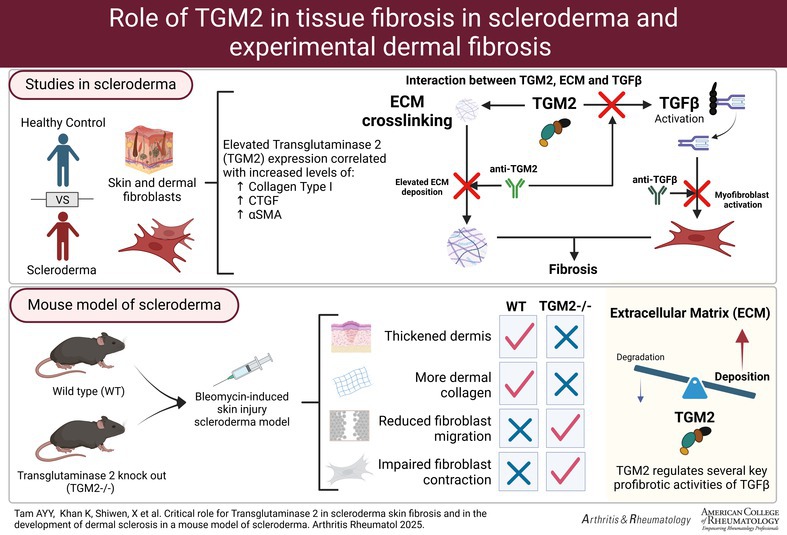

## Graphical abstract

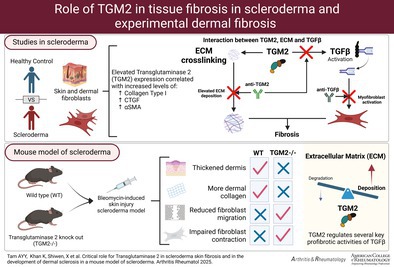

## INTRODUCTION

The connective tissue disease scleroderma (systemic sclerosis [SSc]) is associated with significant morbidity and mortality, with survival dependent on disease subset and organ involvement.^1^, ^2^ Despite some advances in treatments,^3^ there is an unmet need to develop more effective therapies. Elucidation of the pathobiology of scleroderma has been extensively studied^4^, ^5^ and characterized by vasculopathy and persistent inflammation promoting autoimmunity and excessive tissue remodeling and scarring leading to organ‐based replacement fibrosis.^6^, ^7^

In scleroderma and other remodeling diseases, inflammation drives fibrosis, causing excessive scarring.^8^ Fibroblasts play a crucial role in fibrosis, exhibiting functional heterogeneity and undergoing phenotypic switching during fibrosis development. In patients with scleroderma, skin sections reveal abundant fibroblast‐like cells, including myofibroblasts, responsible for excessive production and deposition of extracellular matrix (ECM) proteins.^9^

Transforming growth factor (TGF)‐β is a key driver of fibroblast activation to myofibroblasts in scleroderma.^10^, ^11^ The TGF‐β pathway has been extensively studied at the molecular level and in various fibrotic conditions, including renal and lung fibrosis.^8^, ^12^

Transglutaminases (TGMs) are a family of proteins with structural and functional homology that are enzymatically active and catalyze the formation of post‐translational bonds between proteins.^13^ TGM2, one of the most studied members of the family, is expressed by many cell types and tissues.^14^ In addition to its catalytic function, TGM2 exhibits other functional activities, such as a mediating signaling via G‐coupled protein receptors, as a coreceptor for integrin, a regulator of protein kinases, and an interactor with several proteins presenting adhesion or scaffold‐like properties.^14^, ^15^ Found mainly intracellularly, TGM2 can also be localized to the plasma membrane and found extracellularly.^16^ Within the extracellular environment, TGM2 can become active leading to the extensive crosslinking between many distinct ECM proteins (eg, collagens and fibronectin). Therefore, TGM2 is considered to play a key role in the regulation and homeostasis of the ECM in wound healing^17^ and other critical biologic processes such as cell adhesion, motility, and survival.^14^, ^18^

TGM2 has also been associated with a number of human diseases including inflammatory disorders, renal disease, pulmonary disorders, cancer, cardiovascular disease, and dementia.^14^, ^15^ Indeed, there has been a recent focus on the role of TGM2 in aberrant tissue remodeling and function in scarring and fibrotic diseases.^19^, ^20^, ^21^, ^22^, ^23^ Because of the inherent complex functional activities, there are a number of ways that TGM2 may promote tissue scarring and fibrosis.^15^ For instance, the crosslinking of several common ECM proteins by TGM2 renders them more resistant to degradation, enhancing accumulation and dramatically altering the tissue stiffness and fibroblast cell‐ECM biomechanics.^16^, ^17^ An altered tissue biomechanical environment has been shown to be fundamental in regulating fibroblast function and the myofibroblast phenotype in fibrosis and scleroderma.^11^ Furthermore, TGM2 impacts upon cell survival and enhances cell adhesion and migration,^24^ which are all important aspects of scarring and fibrosis. Importantly TGM2 has been shown to play a crucial role in the activation of TGF‐β1 by crosslinking the large latent TGF‐β1 complex to the ECM initiating release of the active TGF‐β1 dimer.^25^ This has been shown to be a rate‐limiting step in TGF‐β1 activation in fibrotic models.^26^ TGM2 has been shown to be important in studies using in vitro and in vivo models of kidney and lung fibrosis. In the in vitro investigations, TGM2 expression associated with markers of scarring and fibrosis and inhibition of TGM2 reduced myofibroblast formation.^23^ In vivo studies showed that TGM2 knockout (KO) mice exhibited attenuated lung fibrosis following bleomycin challenge^21^ and in a unilateral ureteral obstruction model of chronic kidney disease,^27^ and the addition of a small‐molecule TGM2 inhibitor reduced both glomerular and tubulointerstitial fibrosis in a rodent model of diabetic nephropathy.^20^

A recent study has begun to dissect the intricate role of TGM2 in the activation of SSc fibroblasts and role of TGM2 in fibrosis.^19^ Here we deepen our understanding of TGM2 in the context of scleroderma. Investigations encompassing both skin sections and primary fibroblasts obtained from patients with scleroderma, alongside the bleomycin‐induced skin injury model of sclerosis, sought to unravel the expression and activity of TGM2. These revealed that TGM2 is not only expressed by both control and scleroderma fibroblasts but also exhibits heightened expression levels in scleroderma, thus shedding light on a potentially key role in the pathogenesis of this complex disorder.

In control human dermal fibroblasts, TGM2 could be induced by TGF‐β1 treatment. The presence of a TGM2‐inhibiting antibody^28^ reduced the expression of fibrotic markers in scleroderma cells that were observed with a pan‐TGF‐β neutralizing antibody and an ALK5 inhibitor. TGF‐β bioactivity and Smad2/3 phosphorylation were also reduced in TGM2 inhibited scleroderma cells. *TGM2* KO mice were protected from skin sclerosis. Our data suggest that TGM2 plays a role in skin fibrosis in scleroderma involving interactions between TGM2 and TGF‐β1 in dermal fibroblasts leading to enhanced ECM production and a profibrotic phenotype.

## MATERIALS AND METHODS

### Cell culture

Human dermal fibroblast cultures were established from skin biopsies of patients with diffuse cutaneous SSc (dcSSc), and healthy control (HC) volunteers, as described previously.^29^ All patients met the 2013 American College of Rheumatology/European League Against Rheumatism criteria for SSc.^30^ Informed consent and ethical approval were obtained for all procedures. Fibroblasts were cultured until confluence, and all cell lines were used between passages 2 and 6 for experiments. Confluent monolayers were quiesced in 0.5% bovine serum albumin supplemented Dulbecco's Modified Eagle Medium for 24 hours before lysis for Western blotting. The study was approved by the Health Research Authority, National Research Ethics Service Committee London (Research Ethics Committee reference: 6398). For growth factor treatment, cells were incubated with TGF‐β1 (2 ng/mL; R&D Systems; Cat‐7754‐BH) and incubated for a further 24 hours before being lysed for Western blot analysis. The mature ECM deposited by cultured HC and scleroderma fibroblasts was assessed using a 384‐well immunofluorescence assay.^31^ In some experiments, fibroblasts were cultured in the presence of a TGM2 inhibiting antibody,^28^ a potent and selective TGF‐β Type I receptor/ALK5 inhibitor (10 μM; Tocris Cat1614), or inhibitory antibodies capable of neutralizing all forms of TGF‐β (10 μg/mL; 1D11 Cat ‐ MAB1835).^32^, ^33^

### Immunohistochemistry, activity, and antigen staining for TGM2

Formalin‐fixed 3 μm paraffin sections were used for immunostaining following the blockade of the endogenous peroxidase using 3% hydrogen peroxide. Optimally diluted anti‐TGM2 antibody (1.0 μg/mL)^28^ and anti‐GAPDH (0.2μg/mL; Sigma; Cat ‐SAB4300645) were used in this series. Specificity of staining was confirmed in control sections incubated with an isotype‐matched irrelevant control antibody. Antibodies were detected using the NovoLink polymer kit (Leica Microsystems Cat No: RE7150‐K). Sections were viewed and photographed using a Zeiss Axioscope brightfield microscope using AxioVision software, digitalized using a Hamamatsu NanoZoomer 2.0‐HT Slide Scanner, and visualized using the NDP viewer software. Staining intensity was assessed using semiquantitative assessment of epidermal, dermal, and perivascular staining by an experienced observer blinded to sample identity during scoring. The expression of TGM2 was assessed in whole skin biopsies examining global staining, epidermis, and dermis, infiltrating inflammatory cells when present and in and around vascular structures. Expression was assessed using a semiquantitative grading of the expression, and distribution of staining as assessed by five independent observers trained by a histopathologist and scored between 0 and 3 with 0 (no staining) and 3 (maximal staining) in 0.5 increments. Scoring was performed at ×40 magnification using a global score across the section and three dermal areas for HC (n = 5): dcSSc lesional (n = 5), dcSSc nonlesional (NL; n = 5), established SSc (n = 5), and limited cutaneous SSc (lcSSc; n = 5). Total scores were obtained and average expression values calculated. Graphical data are presented as average staining score ±SD. TGM2 activity was measured by the incorporation of biotinylated cadaverine or T26 peptide,^26^ and TGM2 antigen staining was performed using TGM2 epitope–specific antibodies as described previously.^28^

### Western blot analysis and ECM deposition

Fibroblasts were lysed in radioimmunoprecipitation assay buffer (Sigma) with protease inhibitors (Roche). Total protein concentration was determined by bicinchoninic acid assay (Pierce). Samples were heat denatured under reducing conditions with NuPAGE reducing agent (Invitrogen) and subjected to the NuPAGE electrophoresis system (Invitrogen). Protein bands were transferred onto nitrocellulose membranes (GE Healthcare), which were incubated in block buffer (5% nonfat dry milk, 0.1% Tween‐20 [Sigma] in phosphate‐buffered saline) for 1 hour, followed by incubation at 4°C overnight with primary antibodies against TGM2 (IA12, UCB Pharma), α‐SMA (71 ng/mL, Dako; Cat M0851/1A4), Col‐1 (0.4 μg/mL, Millipore; Cat AB78), CCN2 (0.1 μg/mL, Santa Cruz Biotechnology; Cat SC‐14939), TNC2 μg/mL (Proteintech; Cat 67710‐1), Shad 2 (Cell signaling; Cat 5678), pSmad2/3 (Cell signaling; Cat 8828), and GAPDH (0.2 μg/mL, Abcam; Cat Ab8245/6C5), diluted in block buffer. Blots were developed by incubation with horseradish peroxidase–conjugated secondary antibodies (1:5000, for mouse ‐Cell Signaling; Cat 7076S, for rabbit ‐Cat 7074S). The signal was detected using enhanced chemiluminescence (Pierce) and exposure to Hyperfilm (GE Healthcare). For proteins of similar MW (CCN2, α‐SMA, and GAPDH), equal amounts of protein were loaded, and samples were run simultaneously on separate blots and probed with antibodies, for proteins significantly different in MW (Col‐1, TGM2, TNC2, GAPDH); the same blot was used and probed simultaneously with different antibodies. Densitometry was performed on the bands using Visionworks LS software and displayed in arbitrary densitometry units. For analysis of ECM deposition, dermal fibroblasts from HC and patients with SSc were cultured alone, with TGM2 inhibitory antibody BB7 or IgG control^28^ for 7 days in black wall 384 well imaging plates (BD Biosciences). Cells were removed by lysis with ammonium hydroxide, and deposited proteins were fixed with ice‐cold methanol. ECM accumulation was measured by immunofluorescence staining of individual matrix proteins. Cellomics Arrayscan analysis under the “Cellomics Cell Health” profiling bioapplication used a 10× objective and 2 × 2 binning (1104 × 1104 pixels/field) with “low pass filter” background correction. Matrix was stained with antibodies to fibronectin and collagens I, III, and IV.^31^

### *TGM2* KO mice

All mice used for experiments were aged 4 to 15 weeks at the beginning of the study, including male and female mice. Animals were genotyped by polymerase chain reaction. Mice harboring the *TGM2* null allele have been described previously.^34^, ^35^

### Bleomycin murine model of dermal fibrosis

The bleomycin‐induced murine model was performed by administration of bleomycin (2 units/mL; 0.5mg in 100 μL sterile saline) or saline alone via subcutaneous injection into the dermis as described previously.^36^ After 30 days (treatment time as indicated in each figure), skin tissue was harvested for histology and protein analysis and was fixed with neutral buffered formalin (10%; CellStor).

### Skin histology and advanced microscopy

Mouse dermal samples were fixed in formalin and embedded in paraffin. These tissue blocks were sectioned (4–6 μm) and mounted on glass slides for hematoxylin and eosin (Surgipath and Sigma), Picro Sirius Red (PSR) staining (VWR and Raymond Lab), or Goldner trichrome (MT), for general morphologic analysis and collagen deposition, respectively. The sections were imaged using the NanoZoomer (Hamamatsu) and analyzed using 10× magnification in the NDP View software or imaged using a Zeiss Axio Observer apotome microscope and Axio Cam MR R3 camera and viewed in ZEN Blue microscopy software. For polarized light microscopy,^37^ samples were stained with PSR and observed using a polarized light microscope (Zeiss, Axioskop 2, mot plus). Quantification of collagen fibers under polarized light color threshold analysis was assessed according to published protocols^38^ to assess fibrillar hue and spatial distribution. Data are presented as percentage of stained fibrillar collagen fiber color. For scanning electron microscopy (SEM) of Col‐1 fibrils, samples were prepared by fixation, followed by dehydration, mounting, sputter coated with gold, and images assessed for collagen fiber composition and morphology using a Philips (FEI) 501 Scanning Electron Microscope.^39^ Wound closure and collagen gel contraction assays with fibroblasts were performed and assessed as previously described.^40^, ^41^

### Statistical analysis

Statistical significance to compare groups was calculated by one way analysis of variance or unpaired student two‐tailed *t*‐test, as indicated, using Microsoft Excel or GraphPad Prism V8.43. A value of *P* < 0.05 was considered significant.

## RESULTS

### TGM2 expression in control and scleroderma skin and fibroblasts cultured in vitro

**Figure 1:**
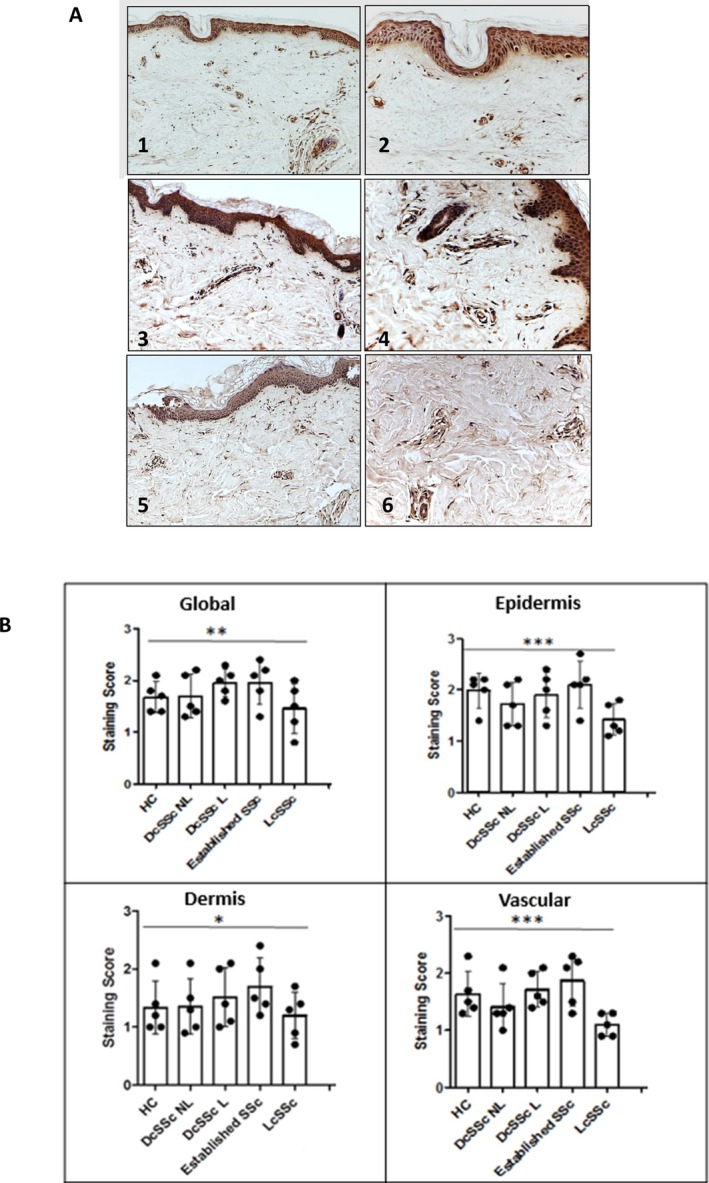
TGM2 expression in control and scleroderma skin and the comparison of TGM2 expression patterns across the scleroderma disease spectrum. (A) Derma biopsies from HC patients with scleroderma (dcSSc; lesional and NL^42^; lcSSc; and established SSc [>5 years duration]) were stained for TGM2 expression using immunohistochemistry (HC: panels 1 and 2; scleroderma NL skin: panels 3 and 4; scleroderma lesional skin: panels 5 and 6; magnification ×10: left; magnification ×20: right). (B) Comparison of expression patterns of TGM2 globally and within specific dermal compartments were made. Expression patterns of cross‐sectional TGM2 and within specific dermal compartments were scored by five blinded observers experienced in examining skin tissue. A semiquantitative grading of the total expression and distribution of staining was employed. Staining was graded between 0 and 3 with 0 (no staining) and 3 (highest staining) in 0.5 increments. Examination was performed at ×40 magnification on five randomly selected areas by each observer per control and disease biopsy. Total scores and average values were determined. Data represent mean staining score ± SD from five biopsy samples. Total score values were used to assess relevance of the differences using statistical significance as determined by *t*‐test *P = 0.05. **P < 0.01, ***P < 0.001. DcSSc, diffuse cutaneous systemic sclerosis; HC, healthy control, lcSSc, limited systemic sclerosis; NL, nonlesional; SSc, systemic sclerosis; TGM2, transglutaminase 2.

**Figure 2:**
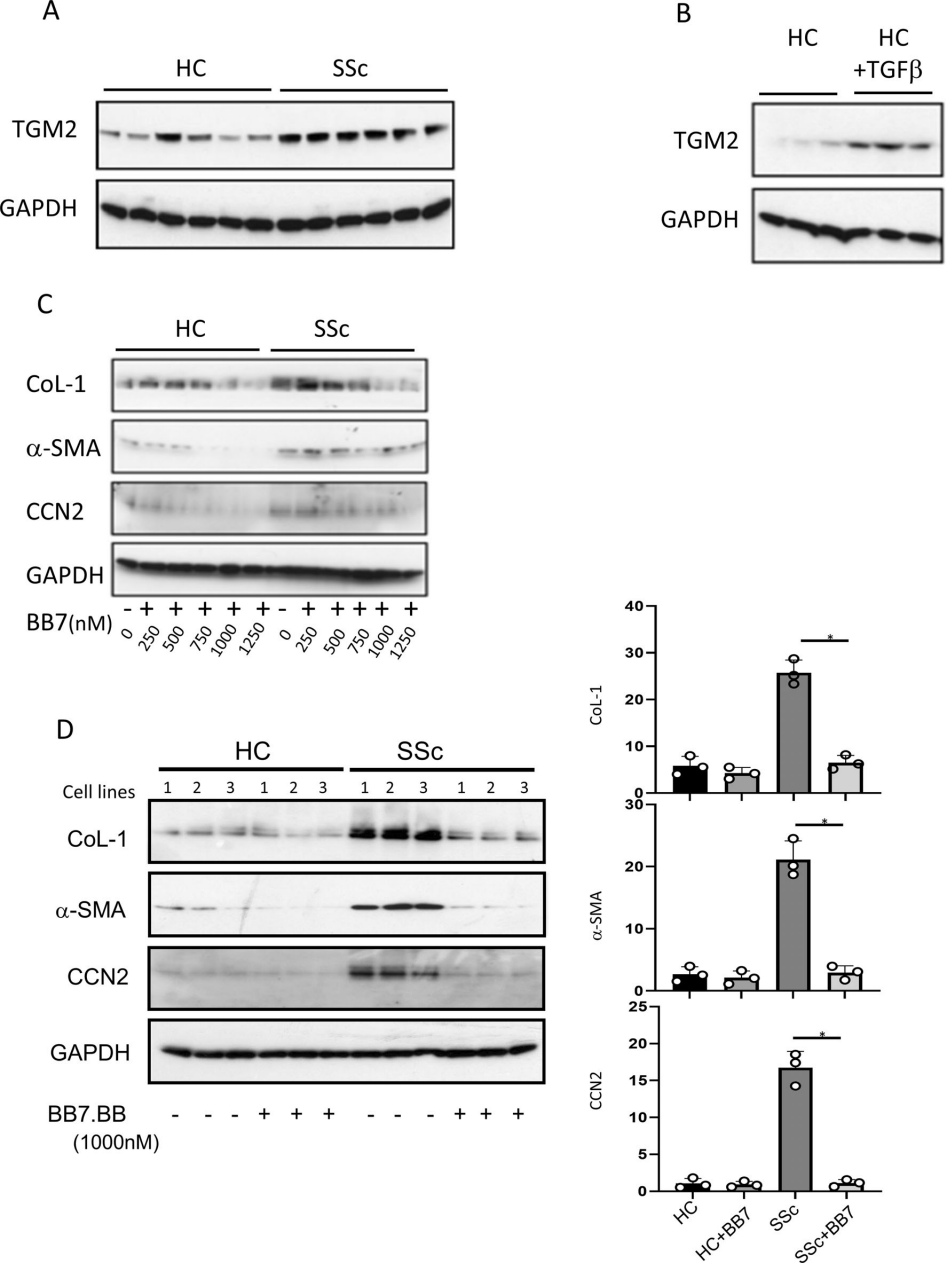
Increased expression of TGM2 by SSc fibroblasts and inhibition of TGM2 attenuates the expression of fibrotic protein markers in SSc dermal fibroblasts in vitro. (A) Expression of TGM2 (IA12 antibody) in primary HC fibroblasts (n = 6) and scleroderma fibroblasts (n = 6) was analyzed by Western blotting and normalized to expression of GAPDH. (B) TGM2 expression in primary HC fibroblasts (n = 3) was determined following fibroblast treatment with TGF‐β1 (4 ng/mL) for 24 hours. (C) Dermal fibroblasts isolated from HC and patients with SSc were cultured with TGM2 neutralizing antibody BB7.BB over a dose response range 0–1250 nM. (D) Survey of three HC and scleroderma fibroblasts cell lines culture in the presence of 1000 nM of BB7.BB. Expression of Col‐1, αSMA, and CCN2 were analyzed by Western blotting and normalized to expression of GAPDH. Densitometry analysis of Western blots (D; right) is shown. Bars show mean ± standard error of the mean. Statistical significance was tested using the *t*‐test, *P < 0.05. HC, healthy control; SEM, scanning electron microscopy; SSc, scleroderma; TGF, transforming growth factor; TGM2, transglutaminase 2.

TGM2 expression in human skin biopsies was investigated using immunohistochemistry with the IA12 antibody. TGM2 staining was observed in all skin compartments (epidermis, dermis, vascular, and inflammatory infiltrates) in both HC and scleroderma samples (Figure 1A). In HC samples, prominent levels were found in the epidermis and vascular compartments, with lower levels in the dermis and inflammatory cells. TGM2 staining was distributed throughout the skin samples, with increased expression observed in inflammatory infiltrates and the dermis. Staining was also visible in secondary dermal structures. TGM2 expression in keratinocytes was evenly distributed in both HC and scleroderma tissue, with a slight decrease in NL samples and in lcSSc (Figure 1B). Elevated expression in dermal fibroblasts was observed in lesional dcSSc, whereas expression levels appeared unchanged in lcSSc and NL dcSSc. Vascular and perivascular staining levels of TGM2 remained unchanged across the scleroderma disease spectrum. Decreased staining levels were observed in lcSSc samples. Elevated TGM2 staining in inflammatory infiltrates was prominent in NL and lesional dcSSc tissue, whereas lcSSc showed unaltered staining in inflammatory infiltrates (Figure 1B). The IA12 antibody used detected an approximately 80Kda protein band of TGM2 in all primary fibroblast lines (Figure 2A). TGM2 expression was higher in SSc fibroblasts compared with normal control counterparts (Figure 2A). Treatment with TGF‐β1 for 24 hours increased TGM2 levels in HC dermal fibroblasts, resembling the levels in SSc dermal cells (Figure 2B). In dermal biopsies, TGM2 insitu‐activity was observed in the epidermis, hair follicles, dermis, and around blood vessels (Supplemental Figure 1A and 1B). Among the eight SSc skin samples examined, one showed increased extracellular TGM2 expression in the dermis, whereas two samples exhibited similar but lower expression levels. The remaining five samples had similar TGM2 staining as healthy volunteers. The three SSc skin biopsies with elevated extracellular TGM2 showed reduced TG activity in the dermis after zampilimab treatment, indicating TGM2‐specific activity (Supplemental Figure 1E and F).

### Inhibition of TGM2 attenuates the expression of fibrotic protein markers in SSc dermal fibroblasts in vitro

To examine the impact of TGM2 inhibition on fibrosis, the levels of Col‐1, CCN2, and αSMA were assessed using specific antibodies and Western blotting (Figure 2C). HC and scleroderma fibroblasts were cultured alone and in the presence of increasing concentrations of TGM2 inhibitory antibody.^28^ Scleroderma fibroblasts exhibited significantly elevated Col‐1, αSMA, and CCN2 compared with HC. The presence of BB7 resulted in a decrease in Col‐1, αSMA, and CCN2 in both cell types, first apparent at a concentration of approximately 500 nM. The BB7 dose‐dependent decrease in Col‐1, αSMA, and CCN2 was more pronounced with the scleroderma cells (Figure 2C). These results were further validated using three different HC and scleroderma cells lines at a dose of 1000 nM, which showed a maximum response in the dose response study (Figure 2D), where the level of expression of the three markers in scleroderma‐derived fibroblasts was found to be significantly reduced to between 70% and 90% of that present in the absence of BB7.

### Deposition of ECM and impact of TGM2 inhibitory and TGF‐β‐neutralizing antibodies

**Figure 3:**
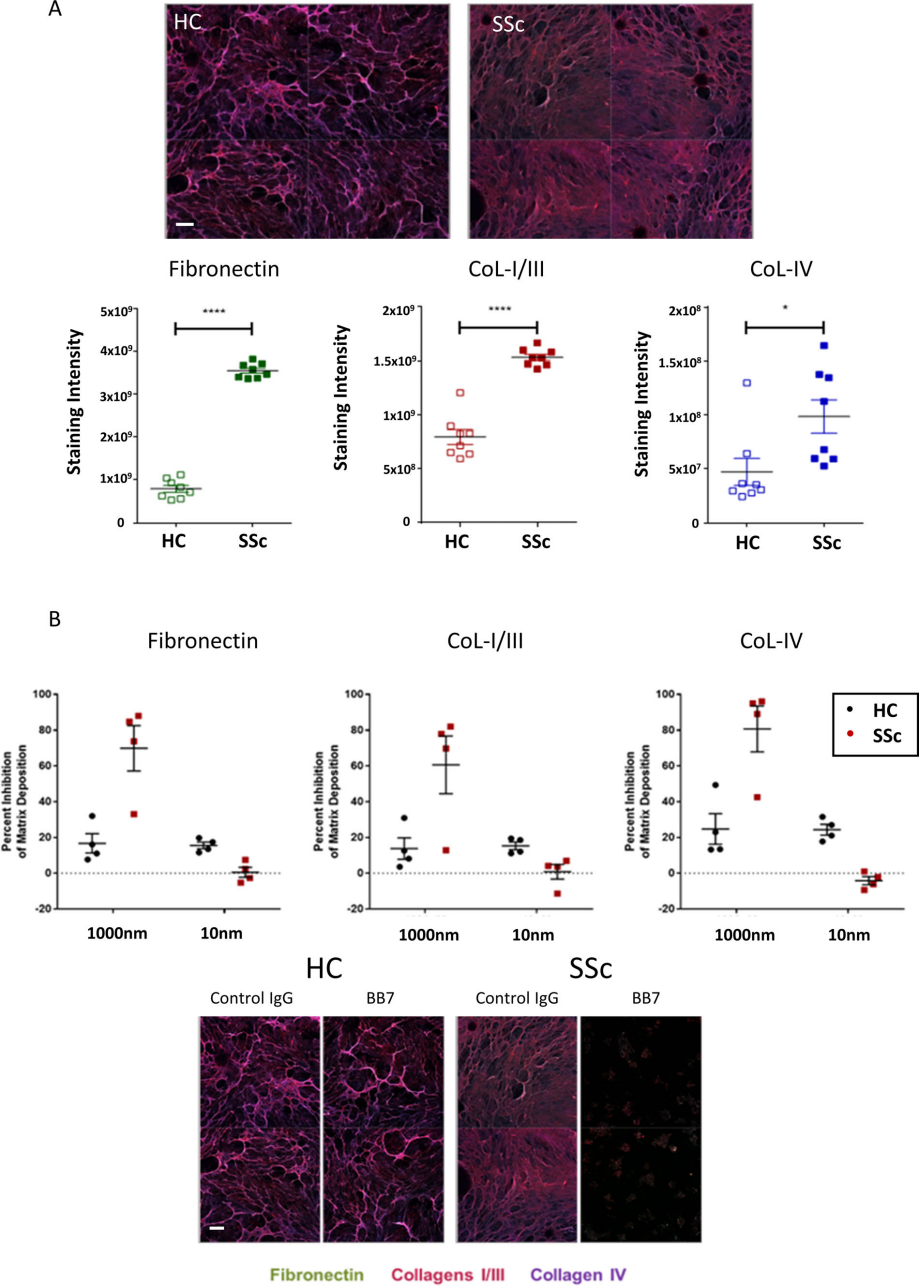
TG2 antibody attenuates extracellular matrix deposition of fibroblasts in vitro. In (A), the readout is mean total intensity for each fluorophore. Data for fibronectin (left), collagens I and III (middle), and collagen IV (right), compare seven cultures derived from HC and seven from patients with SSc. In (B), cells were culture as in (A), but in the presence of BB7, and matrix was stained with antibodies to fibronectin (green) and collagens I, III (purple), and IV (blue). The data show responses from four cultures derived from HC and four from patients with SSc run in 2 independent experiments in the presence of BB7 (10 nm or 1000 nM) relative to 1000nM IgG control and given as percentage reduction in the amount of deposited extracellular matrix.^31^ All images shown are representative merged images. Statistical significance was determined by *t*‐test *P = 0.05, ***P < 0.001. Scale bar = 100 nm. HC, healthy control; SSc, systemic sclerosis. Color figure can be viewed in the online issue, which is available at http://onlinelibrary.wiley.com/doi/10.1002/art.43104/abstract.

The deposition of ECM by primary fibroblast cell lines was measured using a 384 well assay format and high‐content image acquisition using Cellomics software as previously described.^31^ Significantly higher levels of all four ECM components, Fibronectin and collagens types I, III, and IV, were found to be accumulated by SSc fibroblasts compared with the HC cells (Figure 3A). This observation was particularly apparent when examining fibronectin and collagens Type I/III. The presence of the TGM2 inhibitory antibody (BB7;1000nM) resulted in a decrease in the amount of ECM deposited (Figure 3B); this observation was more evident when examining the SSc fibroblasts. Although TGM2 inhibition lowers the ECM levels deposited by SSc cells, it had little impact on the amount of ECM made by normal fibroblasts. At the lower concentration of BB7 (10nM), ECM accumulation in SSc cells was not reduced. Levels of Col‐1 and αSMA were also assessed by immune probing Western blots using specific antibodies and total protein isolated from explanted and cultured fibroblasts from HC and patients with scleroderma (SSc).

**Figure 4:**
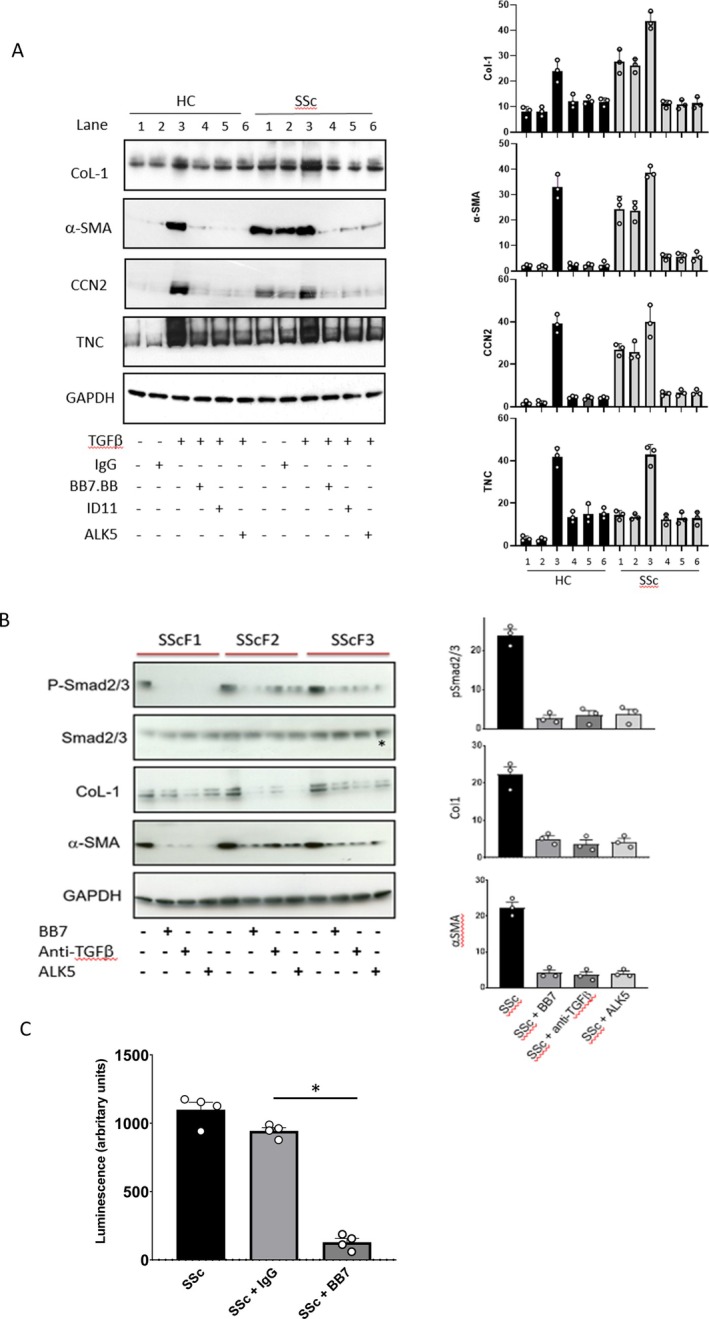
TGM2 inhibition attenuates TGF‐β‐induced expression of fibrotic protein markers and alters Smad 2/3 phosphorylation and TGF‐β levels in control and SSc dermal fibroblasts. (A) Dermal fibroblasts isolated from HC (n = 3) and SSc cell lines (n = 3) were cultured alone or with recombinant TGF‐β1 (4 ng/mL), and then treated with combinations of control IgG, antibody BB7 (anti‐TGM2; 1000 nM), a pan‐TGF‐β blocking antibody (10 ug/mL), or in the presence of a small‐molecule inhibitor of ALK5inh/TGF‐βRI (SB431542; 10uM) for a further 48 hours. Expression of Col‐1, TNC, and αSMA were analyzed by Western blotting; blots shown are representative of n = 3. (B) Levels of SMAD2/3, phospho‐SMAD2/3, collagen I, and αSMA were assessed in SSc cell lines cultured alone or treated with BB7, a pan‐TGF‐β blocking antibody or with the ALK5 inhibitor. (C) Primary SSc dermal fibroblasts were grown in culture and then treated for 48 hours with either of BB7 or control IgG (1000nM). The media was then removed and the level of active TGF‐β measured using the mink lung cell bioassay. Data represent mean ± standard error of the mean for luminescence. *P < 0.05. HC, healthy control; SSc, scleroderma; TGF, transforming growth factor; TGM2, transglutaminase 2. Color figure can be viewed in the online issue, which is available at http://onlinelibrary.wiley.com/doi/10.1002/art.43104/abstract.

Using a panel of TGF‐β‐responsive proteins, in the presence of TGFβ1, addition of anti‐TGM2 antibody (BB7) blocked Col‐1, Tenascin C (TNC), CCN2, and αSMA inductionto a similar level to a pan TGF‐β blocking antibody and ALK5 inhibitor (Figure 4A). Treatment with irrelevant IgG control had no effect on either HC or scleroderma cells. Studies using scleroderma cells revealed that treatment with TGF‐β1, in combination with antibodies BB7 and a pan‐TGF‐β blocking antibody, or in the presence of an ALK5 inhibitor for 48 hours, resulted in a reduction of Col‐1, TNC, CCN2, and αSMA to levels comparable with control cells (Figure 4A).

The impact of BB7 on cellular levels of Col‐1, TNC, CCN2, and αSMA (similar to specific anti‐TGF‐β1 treatments) is intriguing based on blocking just TGM2s extracellular post‐translational modification of the ECM affecting ECM turnover and is more suggestive of a predominant role in TGM2 activation in these cells. This was subsequently investigated by looking at Smad phosphorylation and active TGF‐β1 levels.

### Influence of TGM2 inhibition on Smad 2/3 phosphorylation in dermal fibroblasts

To explore the role of TGM2 in scleroderma fibroblasts, studies were performed to examine whether TGM2 exerted any influence on TGF‐β signaling by examining the Smad pathway. pSmad2/3 levels were readily detected at baseline in all three scleroderma fibroblast lines studied. The elevation in pSmad2/3 was mirrored by high levels of both Col‐1 and αSMA within these cells (Figure 4B). When cultured in the presence of BB7, a pan‐TGF‐β blocking antibody or the ALK5 inhibitor, SB431542, a significant reduction in phosphorylation of Smad2/3, was observed (Figure 4B). Although there was some variation between the three primary SSc cell lines, there was a profound decrease in Col‐1 and αSMA (of between 60% and 80%) in the presence of BB7, a pan‐TGF‐β blocking antibody or the ALK5 inhibitor. This was suggestive of a change in TGF‐β1 driven Smad signaling by blocking TGM2 activity. To confirm this, the levels of active TGF‐β1 were measured in the media of cells exposed to BB7 using the mink lung bioassay and were shown to be reduced by more than 80% (Figure 4C).

### *TGM2* gene deletion attenuates dermal tissue fibrosis in vivo

**Figure 5:**
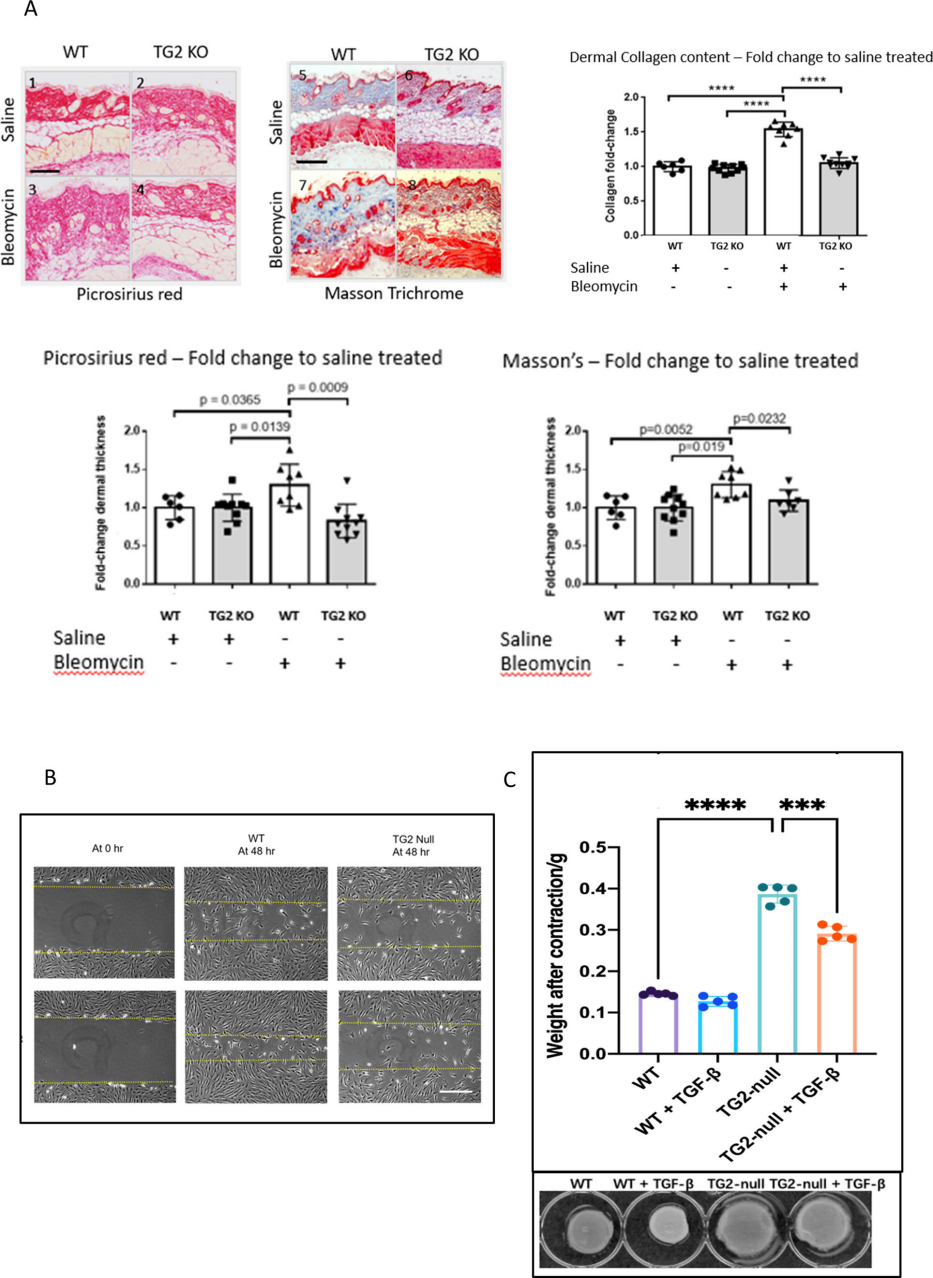
TGM2 gene deletion protects mice from bleomycin‐induced skin fibrosis and impacts upon primary dermal fibroblast functional activities. In (A), skin sections were stained with Masson's Trichrome and picrosirius red and dermal thickness measured using the NanoZoomer NDP software. Three measurements were taken across the skin sections and the averages skin thickness given as fold change relative to saline treated control mice. Stained sections were scanned using a NanoZoomer and viewed with the NDP viewer (×10 magnification). Data are presented as fold change in dermal thickness. *P ≤ 0.05; **P ≤ 0.01; ***P ≤ 0.001. Bar = 200 microns. (B) Primary dermal fibroblasts were culture to confluence, and the monolayers scratched to induce a single injury in the monolayer. A zero time point following wounding (left), scratch repair after 48 hours for WT (middle), and TG2 null (right) fibroblast populations are shown. The upper panel shows migration of fibroblast after 48 hours in the absence and presence (lower panels) of TGF‐β. (C) Dermal biopsies (4 mm) were taken from 6 to 10 mice and snap frozen ready for use. Collagen content was measured using the Sircoll as per the manufacturer's instructions. The Sircoll assay is a colorimetric assay for tissue collagen against a curve of collagen reference standards. Measurements are presented as fold change relative to the saline treated controls ****P = 0.0001. Average collagen content per biopsy were WT/saline (n = 6/27.3 ug), WT/bleomycin (n = 8/41.9ug), TG2 null/saline (n = 10/40.8 ug), and TG2 null/bleomycin (n = 10/43.95 ug). Fibroblast populations derived from explant skin cultures, which were placed with 3D collagen gels (left panel), and their level of contraction was assessed at 48 hours (right panel). Contraction was determined by quantification of gel weight after contraction. Statistical significance was tested by one way ANOVA with Tukey's multiple comparison, *P < 0.05, *** *P* < 0.0001. Scale bar = 200 μm. ANOVA, analysis of variance; KO, knockout; TGM2, transglutaminase 2; TGF, transforming growth factor; WT, wild type. Color figure can be viewed in the online issue, which is available at http://onlinelibrary.wiley.com/doi/10.1002/art.43104/abstract.

In order to investigate the effect of *TGM2* deletion on the development of dermal fibrosis in vivo, we examined the extent of bleomycin‐induced skin remodeling in global *TGM2* KO mice. Figure 5A shows representative skin sections from wild type (WT) and *TGM2* null mice treated with saline and bleomycin. Dermal thickness was measured from both MT‐ and PSR‐stained sections. As shown in Figure 5A, bleomycin treatment caused a significant increase (*P* ≤ 0.05) in the dermal thickness of the WT mice. *TGM2* null mice following treatment with bleomycin showed no changes and were not significantly different from the *TGM2* null mice treated with saline alone. By employing the Sircol assay to assess collagen content, a statistically significant increase (*P* ≤ 0.001) in collagen was observed in the skin of WT mice treated with bleomycin compared with saline alone. In contrast, the *TGM2* null mice exhibited no significant difference or changes in dermal collagen content when treated with saline or bleomycin (Figure 5A). To determine why the *TGM2* KO mice were protected from bleomycin‐induced skin fibrosis, we examined the skin of mice following PSR staining under polarized light and using high resolution SEM. Under polarized light, thick collagen fibers appear red and then progressively thinner through orange, yellow, to green. WT skin appears predominantly red and orange, whereas the *TGM2* KO mouse skin is predominantly green and yellow (Supplemental Figure 2A). This was quantified using Image J software clearly demonstrating a shift toward smaller collagen fibers in the *TGM2* KO (Supplemental Figure 2B). This observation was confirmed using SEM where the thicker “bright” collagen fibers that are most common in WT skin are all but absent in the *TGM2* KO mice (Supplemental Figure 2C).

### Functional assessment of TGM2 deletion in primary dermal fibroblasts

We next performed an in vitro analysis using primary WT and TGM2 KO dermal fibroblasts. Following the injury to the monolayer, although WT mouse fibroblasts were able to effectively repair the wound gap in the injured cell monolayer, at 48‐hours post‐injury only a few *TGM2* null fibroblasts were observed to enter the wound site, exhibiting a defect in migratory capacity (Figure 5B). Furthermore, compared with WT cells, the *TGM2* null fibroblast exhibited a reduced ability to contract 3‐D Col‐1 matrixes (Figure 5C). Addition of TGF‐β1 is able to stimulate a slight increase in contraction in the WT group and significantly increase contraction by *TGM2* null cells. These data indicate that the KO of the *TGM2* gene impacts cell migration and differentiation and that exogenous TGF‐β1 can partially restore and rescue the procontractile behavior.

## DISCUSSION

Our study has shown that TGM2 is highly expressed in scleroderma skin where it is widely distributed and associated with many cell types. Previous studies in renal and pulmonary fibrosis have similarly observed extensive TGM2 expression and activity within a number of cells in fibrotic lesions.^20^, ^21^, ^26^, ^27^ Differences in the temporal expression of TGM2 antigen and activity have also been described^43^ and may be related to the local microenvironment, the allosteric conformational changes in TGM2 that regulate activity and function, and the catalytic dependencies of TGM2 including cofactor availability, sequestration into the extracellular matrisome, and secretion.^14^ Our study did not specifically phenotype the TGM2 expressing cells in the dermis, but suggest that in scleroderma several cell types are contributing to TGM2 expression and release, including fibroblasts. Further studies extending the localization of TGM2 to defined cell types within the skin would be beneficial and could encompass spatial analysis critical to determine cell phenotypes and interactions that impact upon the functional role of TGM2 in skin fibrosis.

The TGM2‐TGF‐β axis has been previously studied and several key reciprocal functional relationships have been elucidated. TGF‐β regulates *TGM2* messenger RNA.^23^, ^25^ Equally, TGM2 is able to modulate TGF‐β levels, activation, and signaling.^25^, ^26^, ^43^, ^44^ Our studies employed primary dermal fibroblast grown in vitro, and in order to minimize activation associated with 2‐D culture, cells were preconditioned in low‐serum to induce quiescence. We also matched HC and SSc fibroblasts for passage and cultured under identical conditions, ensuring differences reflect intrinsic properties. Nonetheless, our experimental conditions appear to have tissue‐culture activated our fibroblasts, as illustrated by the expression of αSMA and CCN2 in our healthy dermal fibroblast populations. We show enhanced levels of TGM2 in scleroderma fibroblasts; observations consistent with previous studies of idiopathic pulmonary fibrosis lung fibroblasts which also showed raised TGM2 levels.^21^ Our findings also confirm and extend previous studies that overexpression of TGM2 is associated with elevated ECM production by fibrotic fibroblasts.^21^, ^23^ Furthermore, our data suggest a direct role for TGM2 as selective inhibition using BB7, an antibody directed toward the catalytic core of TGM2,^28^ which reverses the scleroderma phenotype, reduced the expression of Col‐1, CCN2, fibronectin, and αSMA. This suggests a prominent role for TGM2 in myofibroblast formation.^11^ These studies highlight important interactions between TGM2 and TGF‐β influencing TGF‐β activation and signalling^45^ ^,^ ^46^ and are relevant to our studies exploring fibroblast and myofibroblast formation and function including ECM production, migration, and matrix contraction. Exogenous TGM2 activates lung fibroblasts, which is relevant to idiopathic pulmonary fibrosis.^23^ Furthermore, TGM2 mediated interaction are also known to inhibit Hippo signaling and lead to accumulation of YAP, thereby having the ability to profoundly alter mechano‐transduction.^47^

A number of in vitro studies have highlighted some of the essential requirements needed for the differentiation of fibroblasts into myofibroblasts.^48^ The three key factors widely regarded as required are the presence of growth factors (eg, TGF‐β), an appropriate ECM substrate (such as Fibronectin containing extra domain A/collagen), and the ability of the cells to respond to and develop biomechanical stress. We and others have shown that TGF‐β signaling through the canonical Smad pathway plays a major role in the formation of myofibroblasts.^11^ We demonstrate here that TGF‐β treatment leads to elevated ECM production and deposition and increased myofibroblast transition (as assessed by αSMA expression). The presence of a TGF‐β‐neutralizing antibody or inhibition of TGF‐β receptor signaling using a small‐molecule ALK5 inhibitor prevented increases in both ECM and αSMA expression. Previous studies using different selective ALK5 inhibitors at varying concentrations have reported differing effects on fibroblast αSMA levels and contractile function. These differences in inhibitor selectivity and potency, along with the observations regarding fibroblast activation on different substrates highlighted above, underscore the importance of using appropriate controls, ensuring effective inhibitor potency, and defining fibroblast environments to achieve consistent and robust results.^49^, ^50^

Surprisingly, treatment of TGF‐β‐activated fibroblasts with BB7 also prevented the TGF‐β‐induced increase in Col‐1 and αSMA. Moreover, the presence of BB7 was able to inhibit the levels or activity of TGF‐β secreted by scleroderma fibroblasts, thus attenuating the endogenous Smad signaling observed in these cells thereby preventing the increase in Col‐1 expression and reducing the level of myofibroblast marker αSMA in the cells. These data strongly suggest that in dermal fibroblasts and prominently in scleroderma there is a close biologic interaction between TGM2 and TGF‐β. TGM2 directly influences the level of active TGF‐β and is able to modulate TGF‐β signaling. These data provide evidence to support the notion of a functional relationship between TGM2 and TGF‐β involving coregulation of ECM production and myofibroblast transition. Previous studies have suggested that similar biologic pathways are active in renal disease,^26^ lung fibrosis,^23^ mesenchymal transition involving both endothelial cells^43^ and epithelial cells,^13^, ^15^ and in fibroblast wound healing responses.^18^

To examine the role of TGM2 in vitro and in vivo in tissue fibrosis, a number of studies have been reported and have employed small‐molecule inhibitors,^20^, ^51^ RNA interference,^22^ and *TGM2* null mice.^21^, ^34^, ^35^ As part of our multifaceted approach to studying TGM2 in dermal fibrosis, we used a mouse model of experimentally induced skin fibrosis in the setting of the *TGM2* KO mouse. We studied the extent of tissue remodeling at 28 days following bleomycin treatment, a timepoint giving maximum fibrotic response. We show that *TGM2* null mice were protected from skin fibrosis, exhibiting reduced scar formation when determined histologically and showing no significant increase in dermal collagen content following bleomycin treatment. Our results are in line with other reports examining TGM2 function in several tissues using in vivo models of cardiac,^51^ pulmonary, and renal fibrosis,^21^, ^27^ in which TGM2 inhibition or gene deletion resulted in reduced tissue remodeling and scarring. To further investigate mechanistically why the *TGM2* null mice were resistant to bleomycin treatment and protected from fibrosis, we examined the structure of the dermis in more detail and obtained explanted *TGM2*‐null dermal fibroblasts (fibrotic and controls) in order to study their behavior in vitro and functional activity. Interestingly our work revealed that the skin of *TGM2* null animals was characterized by thinner, newly formed, and less crosslinked collagen fibers. The absence of TGM2 is likely to result in decreased formation of protease‐resistant crosslinks in the dermal ECM fibrils, reduced tissue stiffness, and an increased ECM proteolytic degradation.^52^ Moreover, the ECM reservoir of TGF‐β may also be compromised with less binding of latent TGF‐β binding protein to the matrix.^25^ Together, the more compliant matrix, coupled with depressed bioavailable TGF‐β, may cause a loss in the functional response to stimuli that promote tissue remodeling and fibrosis. Thus, the more rapid turnover of the dermal ECM in the *TGM2* KO mice along with the inability to crosslink the matrix and incorporate and activate latent TGF‐β represents a plausible hypothesis that underlies the failure to progress scar formation and develop fibrosis.

Our studies note that *TGM2* gene deletion imparts a profound influence on fibroblast function, with a severe impact on cell migration and the ability of *TGM2* null cells to remodel Col‐1 3‐D matrixes. The significantly reduced migration of these fibroblasts following scratch wounding and inability to effectively contract collagen gels suggests alterations in cell adhesion, attachment and motility, and an impairment in the transition of fibroblasts to their activated contractile myofibroblasts counterparts. In support of these observations, cell‐surface TGM2 has been reported to be essential for the organization of cell adhesion foci involving integrins, fibronectin, and matricellular proteins and is therefore pivotal for fibroblast adhesion and migration.^13^, ^14^, ^15^, ^35^ TGM2 is also known to facilitate the regulation of signaling pathways that govern stress fiber formation and myofibroblast differentiation,^53^, ^54^, ^55^, ^56^ and which results in the expression of potent profibrotic genes such as CCN2 and collagen.^54^ These studies are consistent with what we show in our current study, that the absence of TGM2 expression by fibroblasts results in several key functional deficits that are crucial to scarring and fibrosis, thereby increasing our understanding of the role of TGM2 in fibrogenesis.

It is important to note that these current studies, along with the recent report by Zhou et al,^19^ are the first to begin to explore in detail and understand the role of TGM2 in scleroderma. Although we find a substantial agreement between the findings of Zhou et al and our own in the current study, especially evident in 3‐D and in vivo models, there are notable disparities with the 2‐D cell culture systems. Our study uses low passage fibroblasts obtained from patients with early dcSSc derived by whole tissue explant culture and not enzymic‐treatment yielding cells with a pronounced fibrotic phenotype and HCs, an experimental design that contrasts with that of Zhou et al, as their reported data in 2‐D primarily involves TGF‐β‐treated fibroblasts cultured in 2‐D, enabling direct comparisons that are inherently limited. Plausible explanations for this disparity are that 2‐D culture environment is likely to induce alterations in fibroblast behavior, although this effect would likely impact both SSc and HC cells alike and the potential confounding impact of the passage dependency phenomenon, wherein the disease phenotype diminishes with prolonged in vitro culture. Our data (unpublished) corroborate this phenomenon, notably illustrating a marked reduction in TGM2 expression and responsiveness to inhibition with advancing passage. The observed loss of disease phenotype in 2‐D culture may potentially be mitigated or unmasked when cells are cultured within a 3‐D environment. Additionally, considering the inherent plasticity and adaptability of fibroblasts, further investigation into the impact of culture conditions and passage number on disease phenotype maintenance is warranted, particularly in this context. Nonetheless, together these data provide strong evidence for the involvement of TGM2 in dermal fibrosis in scleroderma through the regulation of TGF‐β and the reciprocal modulation by TGM2 of TGF‐β functional activity, thus revealing that there are several ways by which TGM2 can promote fibrosis. Although this current study did not assess scleroderma lung, TGM2 is expressed by pulmonary fibroblasts.^21^ In view of the previous persuasive data concerning the role of TGM2 in interstitial lung diseases,^21^, ^23^ it is likely that TGM2 would exert an important influence in the pulmonary complications in scleroderma. The availability of a human specific monoclonal antibody for TGM2^28^ provides extra impetus toward the potential use of TGM2 blockade in scleroderma and would significantly add to the increasing number of antifibrotic therapies available or currently under investigation for this scleroderma.

## AUTHOR CONTRIBUTIONS

All authors contributed to at least one of the following manuscript preparation roles: conceptualization AND/OR methodology, software, investigation, formal analysis, data curation, visualization, and validation AND drafting or reviewing/editing the final draft. As corresponding author, Dr Abraham confirms that all authors have provided the final approval of the version to be published, and takes responsibility for the affirmations regarding article submission (eg, not under consideration by another journal), the integrity of the data presented, and the statements regarding compliance with institutional review board/Declaration of Helsinki requirements.

## ROLE OF THE STUDY SPONSOR

UCB had no role in the study design or in the collection, analysis, or interpretation of the data, the writing of the manuscript, or the decision to submit the manuscript for publication. Publication of this article was not contingent upon approval by UCB.

## Supporting information


**Disclosure form**.

Supplementary Figure 1:

Supplementary Figure 2:
