# Supplementary figures and images for: Critical Role for Transglutaminase 2 in Scleroderma Skin Fibrosis and in the Development of Dermal Sclerosis in a Mouse Model of Scleroderma
Source: Arthritis Rheumatol. 2025 May 19;77(7):914–28. doi: 10.1002/art.43104 (PMC12209749; doi:10.1002/art.43104)

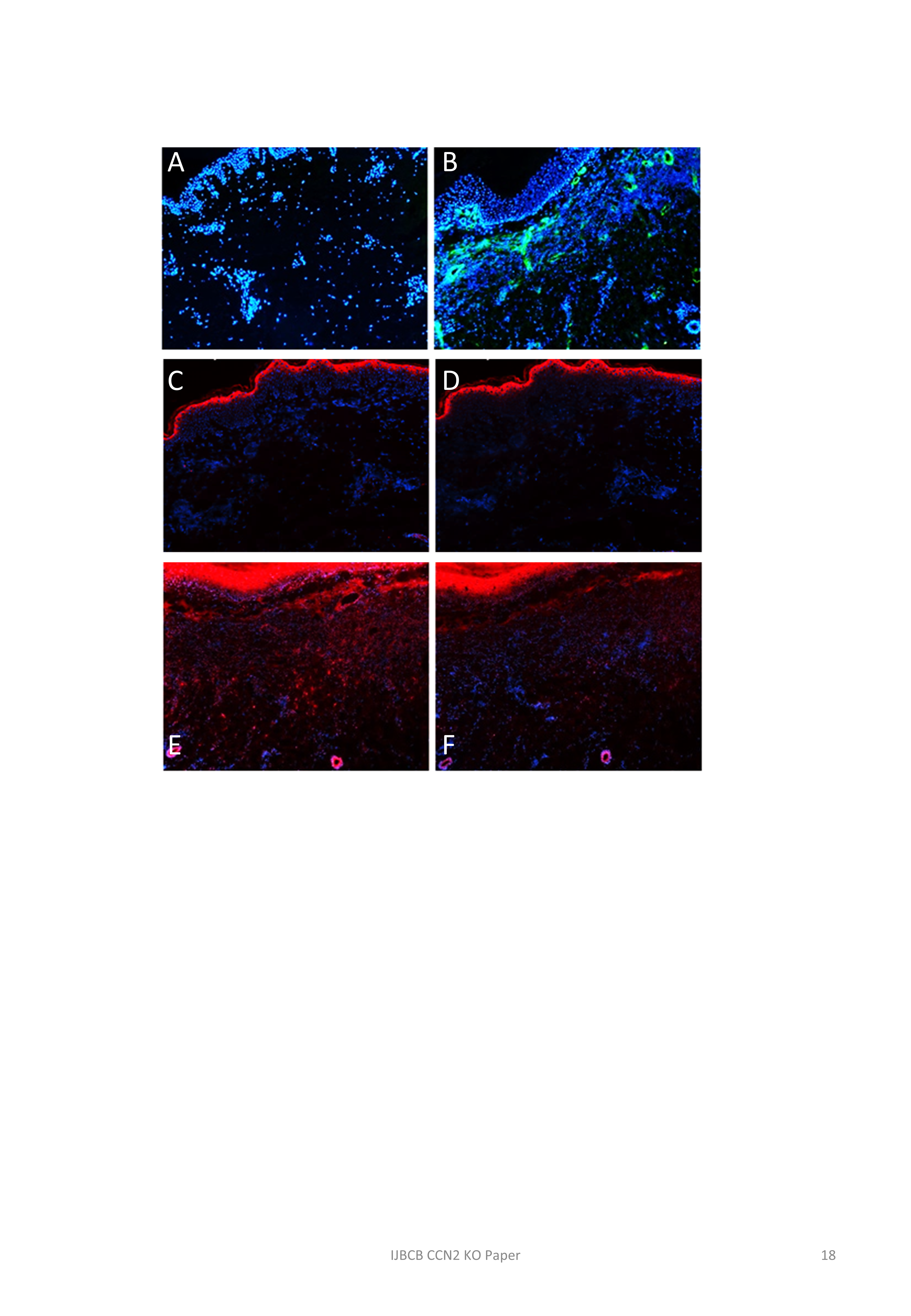

Supplement: Supplementary file 2 — Supplementary Figure 1: [file ART-77-914-s002.tif]

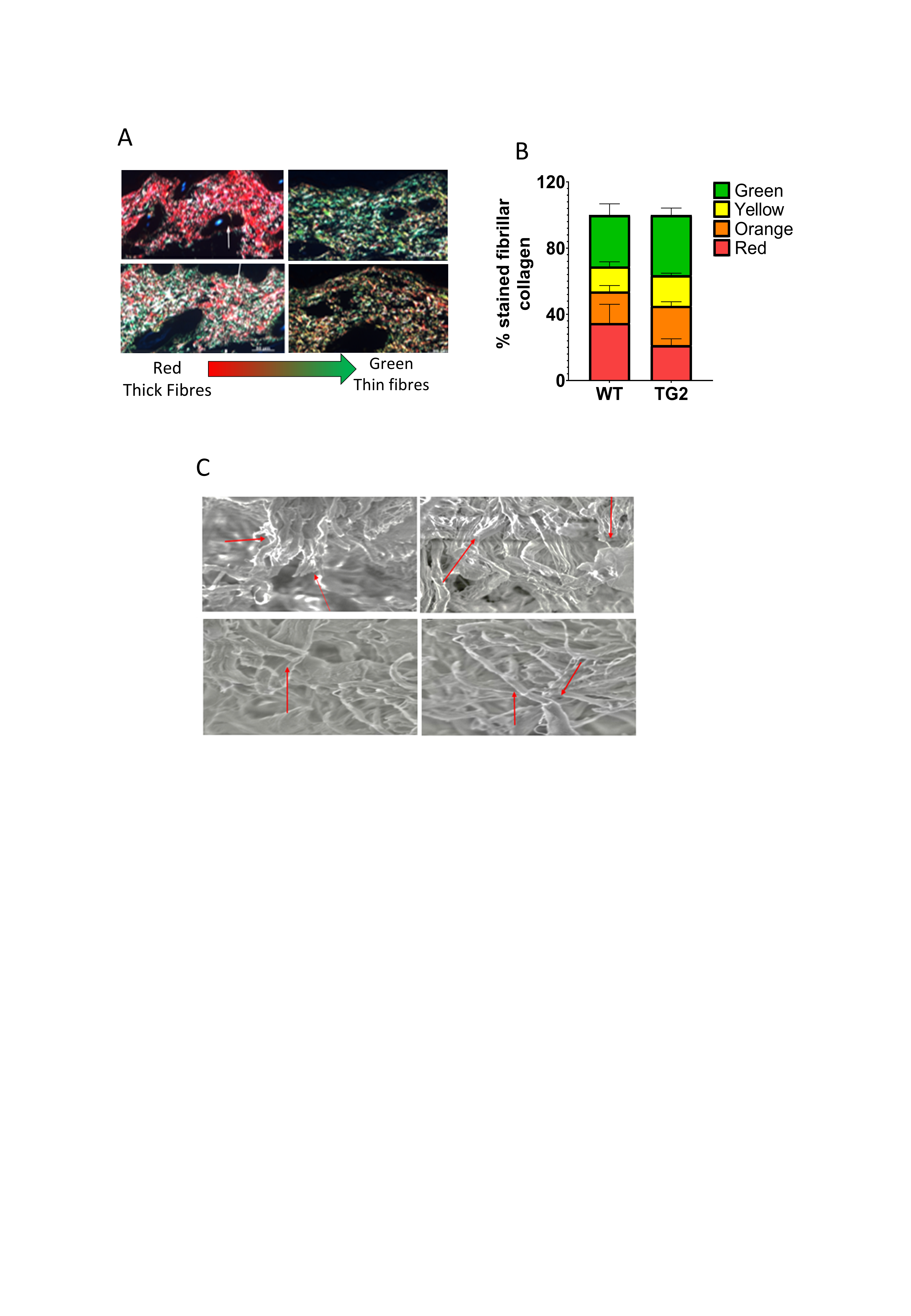

Supplement: Supplementary file 3 — Supplementary Figure 2: [file ART-77-914-s001.tif]
